# Multiple Impacted Supernumerary Teeth in a Non-syndromic Class III Malocclusion Patient: A Case Report

**DOI:** 10.7759/cureus.102941

**Published:** 2026-02-04

**Authors:** Wissam El Hazzat, Nawal Bouyahyaoui, Fatima Zaoui

**Affiliations:** 1 Department of Dentofacial Orthopedics, Faculté de Médecine Dentaire de Rabat, Université Mohammed V de Rabat, Rabat, MAR

**Keywords:** class iii malocclusion, cone-beam computed tomography (cbct), non-syndromic, orthondontic treatment, supernumerary teeth

## Abstract

Supernumerary teeth are considered a numerical dental anomaly characterized by an excess of teeth. They may appear as single, double, or, more rarely, multiple teeth and can be associated with various syndromes.

A 21-year-old male patient presented to the orthodontic department at the Dental Consultation and Treatment Center in Rabat, Morocco, seeking care for esthetic concerns.

Clinical examination revealed a Class III malocclusion with crowding, the absence of tooth 44, and arch asymmetry. Panoramic radiography revealed the impaction of tooth 44 along with five additional supernumerary teeth: two supplementary canines in the maxillary jaw located between the lateral incisors and canines bilaterally, one paramolar situated between the left mandibular premolars, and two paramolars in the right mandibular premolar region.

The supernumerary teeth, as well as teeth 14, 24, and 34, were extracted before initiating orthodontic treatment. A Class I occlusion was achieved.

The incidental discovery of multiple supernumerary teeth with consequences on dental arches in patients with no systemic conditions underscores the importance of panoramic radiographs during the mixed dentition period for preventive purposes.

## Introduction

Supernumerary teeth may prompt consultation if they erupt in the arch or cause crowding, malocclusion, delayed eruption, or disturbances in eruption. However, in 75% of cases, they remain asymptomatic and are incidentally discovered during routine clinical and radiographic examinations [1,2].

The prevalence of non-syndromic multiple supernumerary teeth ranges from 0.1% to 3.8%, with higher prevalence in the permanent dentition and higher frequency in the Asian population. Also known as hyperdontia, this anomaly involves an excess number of teeth. The crown of a supernumerary tooth may mimic normal tooth morphology, appear conical (mesiodens), or be atypical, as seen in odontomas. These teeth can present in normal or ectopic positions and may follow various orientations [3,4].

Hyperdontia is often part of a syndrome such as cleidocranial dysplasia or Gardner syndrome. In contrast, the presence of multiple supernumerary teeth in non-syndromic patients has been described only sporadically, mainly through isolated case reports. Although the exact etiology remains unclear, several hypotheses suggest the involvement of genetic susceptibility, epigenetic regulation, and local environmental factors influencing dental lamina activity [1-5].

Management depends on their effect on the dental arches, impaction status, and presence of associated dysmorphoses. Treatment options range from abstention to extraction, with or without orthodontic intervention. Cone-beam computed tomography (CBCT) plays a crucial role in the diagnosis and management of multiple supernumerary teeth by providing a three-dimensional assessment of tooth position, morphology, and relationships with adjacent anatomical structures. CBCT is particularly valuable in complex cases to guide therapeutic decision-making and reduce the risk of iatrogenic complications [6].

This article presents a rare non-syndromic case involving five impacted supernumerary teeth and an impacted tooth 44, associated with Class III malocclusion and skeletal discrepancy.

## Case presentation

A 21-year-old male patient presented to the dentofacial orthopedics department in Rabat, Morocco, for evaluation due to significant dental crowding and ectopic canines. The patient had no history of dental or facial trauma, and family history revealed no consanguinity or other genetic factors influencing the diagnosis.

Clinically, the patient had a symmetrical square-shaped face, flat cheekbones, balanced facial thirds, and a slightly convex profile with maxillary retrocheilia.

**Figure 1:**
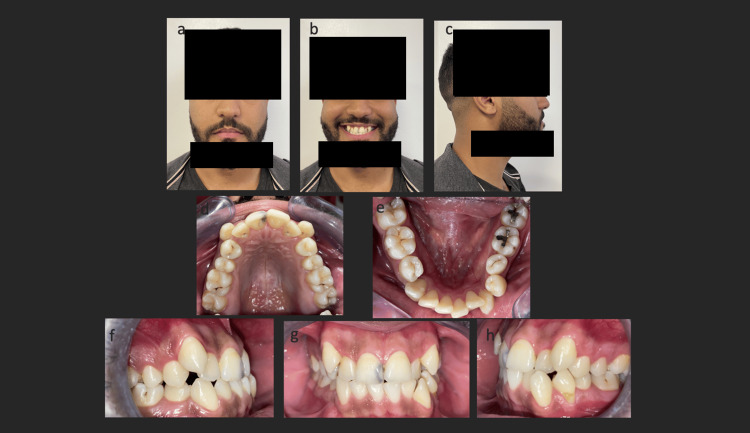
Extraoral (a, b, c) and intraoral (d, e, f, g, h) photographs at treatment initiation showing Class III occlusion, ectopic canines, missing tooth 45, and notable esthetic discrepancy.

Intraorally, significant maxillary and mandibular incisor-canine crowding, edge-to-edge occlusion, left canine and molar Class III, and right Class II canine and Class III molar relationships were observed due to the absence of tooth 44 (Figure 1).

**Figure 2:**
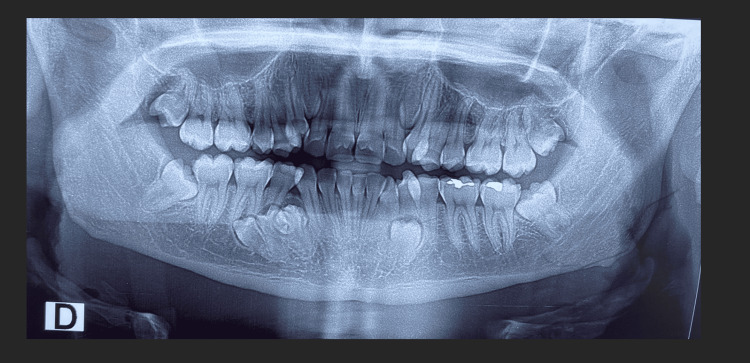
Panoramic radiograph showing impaction of tooth 44 and five supernumerary teeth: two supplementary canines between the lateral incisors and canines bilaterally, one left paramolar between the left mandibular premolars, and two right mandibular paramolars.

**Figure 3:**
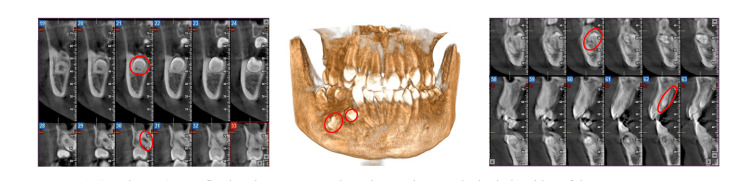
CBCT images illustrating multiple impacted supernumerary teeth. CBCT images show six impacted teeth, including five supernumerary teeth. Two vertically impacted teeth are observed in the maxilla. In the mandible, one impacted premolar is present in the left premolar region, while three impacted premolars are located in the right premolar region, two of which are horizontally oriented. The left and right panels show sequential sagittal CBCT slices of the anterior and posterior regions, while the central panel presents a 3D volume-rendered reconstruction of the jaws. Sagittal CBCT sections reveal the presence of five impacted supernumerary teeth, located in different regions and orientations, clearly identified and outlined in red. The 3D CBCT reconstruction confirms the spatial distribution, and anatomical relationships of these supernumerary teeth within the jaws. Additionally, an impacted mandibular right first premolar (tooth 44) is visible and circled in red on the sagittal CBCT images, showing an abnormal position within the mandibular bone. CBCT, cone-beam computed tomography; 3D, three-dimensional.

Radiological assessment (panoramic and cone-beam CT) incidentally revealed six impacted teeth, five of which were supernumerary. Two maxillary supernumeraries were vertically positioned between the lateral incisors and canines bilaterally. In the mandible, one premolar was impacted in the left premolar region, while three premolars were present in the right premolar region, two of which were horizontally oriented (Figures 2, 3).

A comprehensive orofacial examination was performed to rule out syndromic conditions. Facial symmetry, skin, hair, nails, and systemic features were all within normal limits.

Cephalometric analysis revealed a skeletal Class III relationship, with an SNA (Sella-Nasion-A point angle) of 79°, an SNB (Sella-Nasion-B point angle) of 80°, an ANB (A point-Nasion-B point angle) of -1°, and a Wits appraisal of -3 mm, reflecting a mild skeletal Class III pattern with a maxillo-mandibular discrepancy that measured 18 mm. These findings correlate with the intraoral observation of edge-to-edge anterior occlusion and the asymmetrical molar and canine relationships (Table 1).

**Table 1:** Pre- and post-treatment with cephalometric values according to Steiner and Tweed. SNA: the maxilla (point A) is related to the cranial base (SN); SNB: the mandible (point B) is related to the cranial base (SN); ANB: the difference between SNA and SNB; AoBo: represents the projection of the points A and B on the occlusal plane; I/to NA: maxillary incisor version; I/to NA (mm): maxillary incisor position; i/ to NB: lower incisor version; i/ to NB (mm): lower incisor position; GoGnSN: mandibular plane to cranial base; FMIA: the long axis of the mandibular incisor to the Frankfort plane; FMA: Frankfort mandibular plane angle; IMPA: the lower incisor's axial inclination to the mandibular plane. [7,8].

Cephalometric parameter	Reference value	Pre-treatment cephalometric value	Post-treatment cephalometric value
SNA (°)	82	79	80
SNB (°)	80	80	80
ANB (°)	-2+2	-1	0
AoBo (mm)	0+-2	-3	-1
I/NA (°)	22	21	30
ItoNA (mm)	4	0	6
i/NB (°)	25	21	27
itoNB (mm)	4	3	4
GoGn/SN (°)	32	30	33
FMIA (°)	67	66	65
FMA (°)	25	24	24
IMPA (°)	88	90	91

The combination of clinical, radiographic, and cephalometric findings supported the diagnosis of skeletal Class III with a normodivergent profile, associated with dento-maxillary disharmony (DDM), impaction of tooth 44, and the presence of five additional impacted supernumerary teeth in both maxilla and mandible.

Management was interdisciplinary in collaboration with the surgical dentistry department. The surgeon extracted all six impacted teeth and teeth 14, 24, and 34 for orthodontic reasons. The patient was also referred for necessary restorative dental care, and the orthodontic treatment was sought after full recovery.

**Figure 4:**
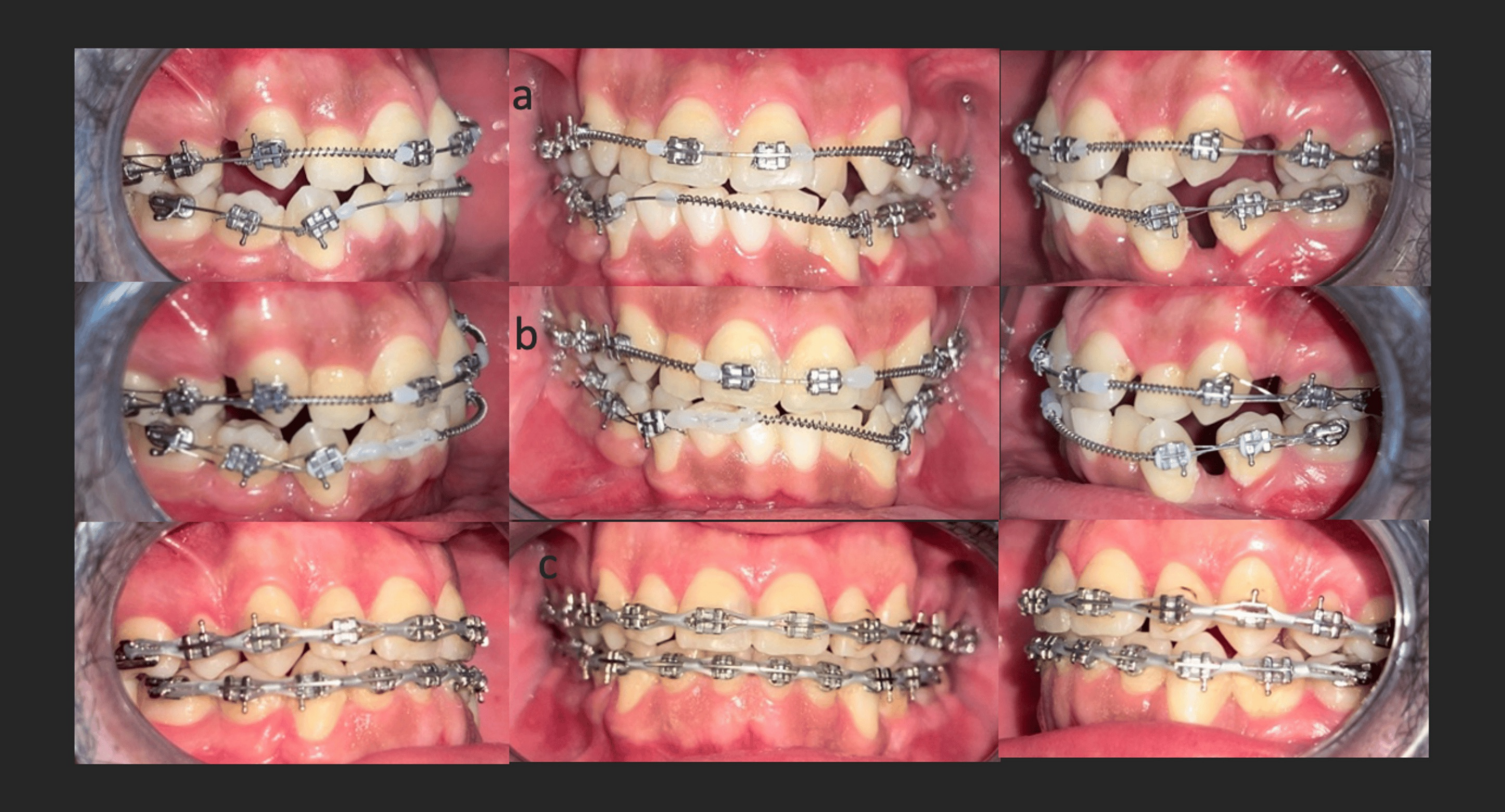
Intraoral photographs showing various treatment stages: a: alignment and leveling, b: incisor-canine retraction, and c: finishing.

Conventional orthodontic brackets were used. Alignment and leveling were achieved with a sequence of NiTi archwires (0.014, 0.016, 0.018, 17×25). Mandibular incisor alignment was delayed to prevent labial tipping and loss of the anterior occlusal key (Figure 4).

Subsequent incisor-canine retraction was performed using 17×25 and 18×25 stainless-steel wires. Class III elastics were introduced at the end of the leveling stage to improve intercuspation.

**Figure 5:**
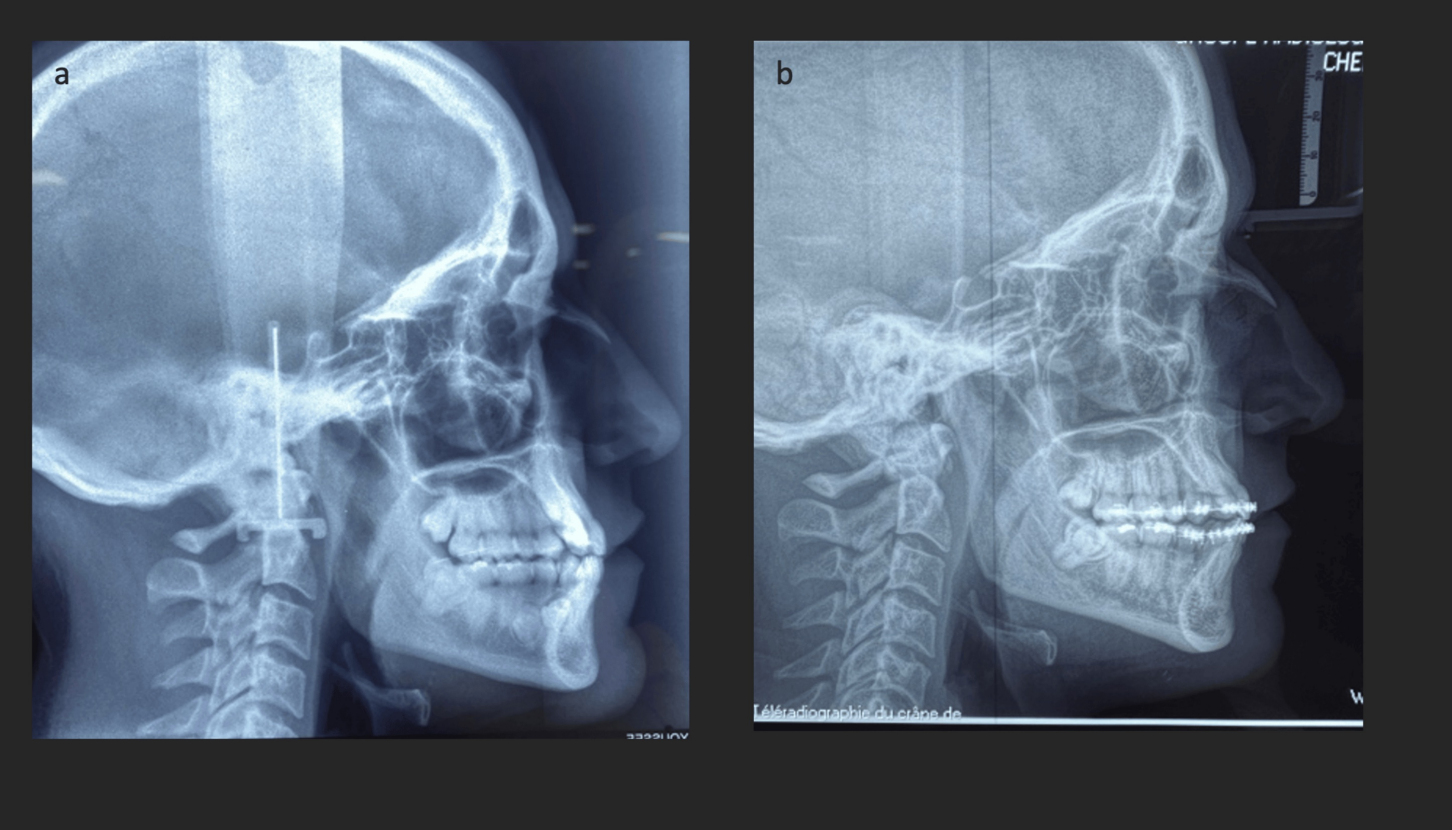
Lateral cephalometric radiographs; a: pretreatment and b: post-treatment.

**Figure 6:**
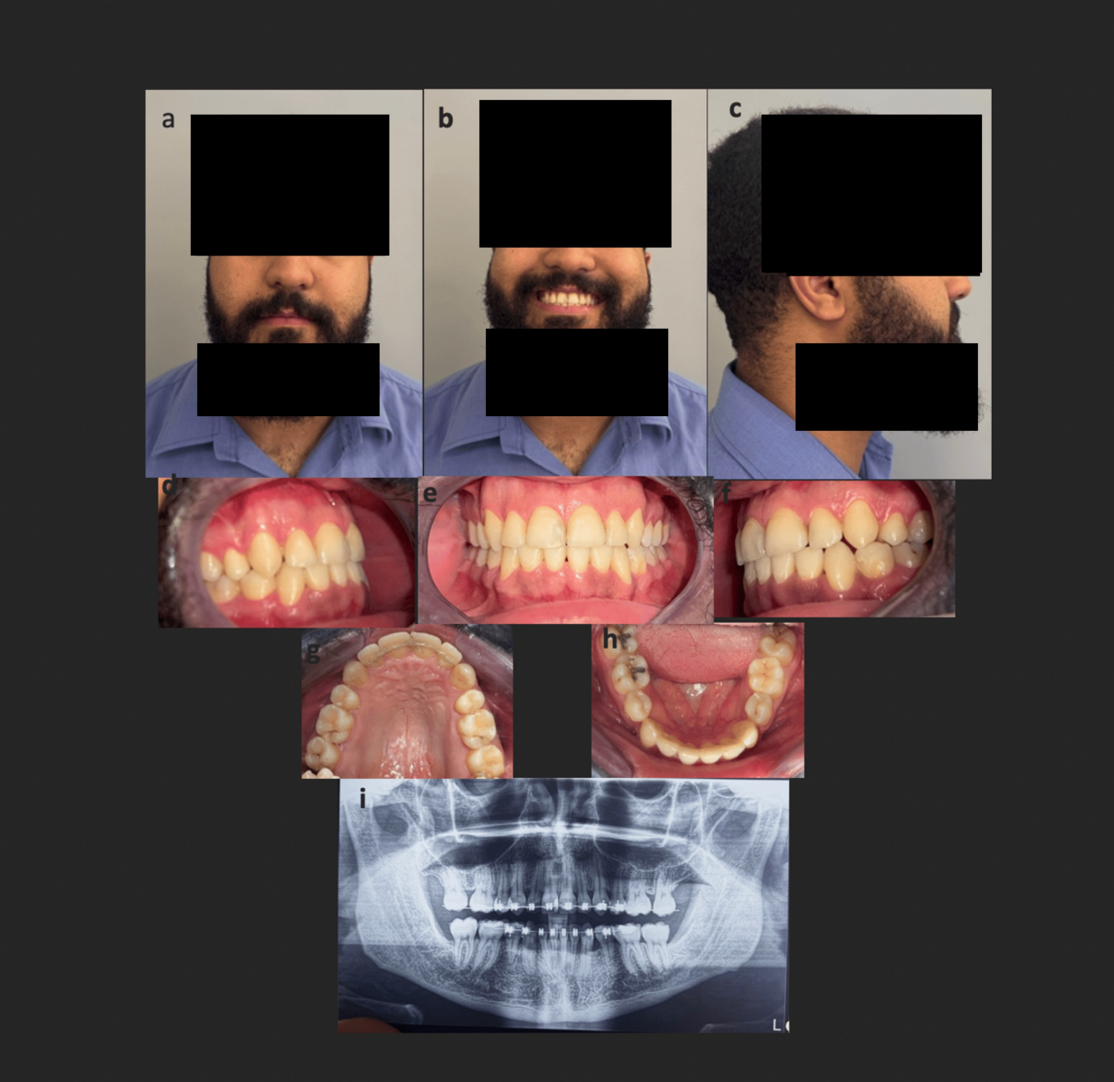
Extraoral (a, b, c) and intraoral (d, e, f, g, h) images and panoramic radiograph (i) showing clinical outcomes after 20 months of treatment. The extraction of the wisdoms was carried out given their unfavorable axes for the eruption.

Treatment lasted 20 months, and fixed retainers were placed in both arches, along with a removable maxillary retainer (Figure 5, panel b; Figure 6).

Evaluation of the panoramic radiographs before (Figure 2) and after treatment (Figure 6) demonstrated significant improvement in dental alignment, adequate root parallelism, and stable periodontal conditions, with no evidence of root resorption or other radiographic complications. Pre-treatment lateral cephalometric analysis revealed sagittal and dentoalveolar discrepancies, including altered incisor inclinations and an unfavorable skeletal relationship. Following treatment, the post-treatment cephalometric assessment (Table 1) showed marked improvement in sagittal jaw relationships, correction of incisor angulations, and enhanced soft-tissue profile harmony. These radiographic and cephalometric changes, in addition to the clinical outcomes, indicate an orthodontic correction with stable skeletal, dental, and facial outcomes.

## Discussion

This report presents a rare case of multiple non-syndromic supernumerary teeth in both jaws, discovered incidentally during a routine orthodontic consultation.

Although the supernumerary teeth had minimal impact on the dental arches, there was a slight deviation of interincisal midlines and asymmetrical occlusal relationships due to the impaction of tooth 44, likely caused by adjacent supernumerary paramolars.

According to Steiner’s analysis, the DDM measured 18 mm, indicating a significant discrepancy between the available space and the space required for proper alignment of the teeth [7]. This assessment justified the extraction of supernumerary teeth and selected premolars to allow controlled tooth movement, midline correction, and optimal occlusion, while minimizing the risk of root resorption or tipping.

The patient underwent extraction of the supernumerary teeth as well as teeth 14, 24, 34, and the impacted 44 to address orthodontic concerns. After a healing period of six months, orthodontic treatment was initiated with alignment and leveling, followed by space closure and final occlusal finishing; to manage such skeletal Class III discrepancy, intermaxillary traction has been used for its hard- and soft-tissue effects [9]; the treatment was completed in 20 months, and Class I canine and molar occlusion was achieved post-treatment.

Such cases are rare; supernumerary teeth are typically single (65.8-80.5%), double (14.5-27.7%), and multiple in only 0.6-8% of cases. Multiple supernumerary teeth are usually associated with syndromes like Gardner’s syndrome, cleidocranial dysplasia, orofacial clefts, Crouzon syndrome, Franceschetti syndrome, Hallermann-Streiff, Down syndrome, Noonan syndrome, Rubinstein-Taybi, Zimmermann-Laband, Fabry disease, Ellis-Van Creveld, and Ehlers-Danlos syndromes [1,3-6]. Unlike syndromic cases, multiple supernumerary teeth occur without systemic anomalies. Genetic factors are thought to contribute to hyperdontia, including mutations in genes regulating dental development, although environmental factors may also play a role [10].

Supernumerary teeth may erupt in the arch or ectopically, causing dentoalveolar disharmony or occlusal issues. They may remain impacted in atypical locations such as the nasal cavity, mandibular border, or maxillary sinus. Incidental discovery, as in this case, is not uncommon. However, associated anomalies like delayed eruption of permanent teeth, especially central incisors in the presence of mesiodens or odontomas, may necessitate consultation. Other complications include retained deciduous teeth, crowding, or follicular cysts [11-14].

Epidemiologically, the male gender is more frequently affected, and mandibular involvement is more common [13]. In this case, both jaws were involved. Maxillary supernumeraries were conical, and mandibular ones resembled premolars with atypical roots, as shown by panoramic and cone beam imaging [15]. Panoramic, lateral cephalometric, and CBCT imaging allowed precise identification of the supernumerary teeth, directly informing the treatment plan.

The impaction orientation was vertical in the premaxillary region and for the left paramolar, oblique for tooth 44, and transverse for the adjacent paramolars. In general, hyperdontia presents with vertically, transversely, or even invertedly impacted teeth [16].

Management options include abstention, extraction with or without orthodontic treatment, depending on the severity of their impact on occlusion and arch integrity [2-4]. In this case, extraction followed by orthodontic correction was chosen. Treatment challenges included space closure due to root divergence and midline correction related to unilateral impaction of the lower premolar. Extraction of third molars was also planned due to their unfavorable angulation. Fewer consequences on the arches could have been detected if the supernumerary teeth had been diagnosed at an early age and the treatment had been undertaken earlier. Early detection of supernumerary teeth could have reduced space issues and shortened treatment duration, highlighting the importance of timely diagnosis and intervention to minimize complications and optimize orthodontic outcomes.

## Conclusions

This case report presents the orthodontic management of a non-syndromic Class III malocclusion associated with multiple supernumerary and impacted teeth. CBCT facilitated precise diagnosis and guided treatment planning, while preventive panoramic radiography proved valuable for early detection. Favorable occlusal, functional, and esthetic outcomes were achieved; however, these results should be interpreted with caution due to the inherent limitations of a single-case report. Postoperative considerations, including potential complications, were carefully monitored and managed. Despite these limitations, this case contributes to the existing literature and underscores the importance of early diagnosis and comprehensive assessment in similar clinical scenarios. Future studies involving larger cohorts are warranted to further validate these findings.
